# Performance Assessment of Kernel Density Clustering for Gene Expression Profile Data

**DOI:** 10.1002/cfg.290

**Published:** 2003-06

**Authors:** Guoping Shu, Beiyan Zeng, Yiping P. Chen, Oscar H. Smith

**Affiliations:** Reid Research Centre Pioneer Hi-Bred International Inc. DuPont Agriculture and Nutrition 7300 NW 62nd Avenue, PO Box 1004 Johnston IA 50131 USA

## Abstract

Kernel density smoothing techniques have been used in classification or supervised learning of gene expression profile (GEP) data, but their applications to clustering or unsupervised learning of those data have not been explored and assessed. Here we report a kernel density clustering method for analysing GEP data and compare its performance with the three most widely-used clustering methods: hierarchical clustering, K-means clustering, and multivariate mixture model-based clustering. Using several methods to measure agreement, between-cluster isolation, and withincluster coherence, such as the Adjusted Rand Index, the Pseudo *F* test, the r^2^ test, and the profile plot, we have assessed the effectiveness of kernel density clustering for recovering clusters, and its robustness against noise on clustering both simulated and real GEP data. Our results show that the kernel density clustering method has excellent performance in recovering clusters from simulated data and in grouping large real expression profile data sets into compact and well-isolated clusters, and that it is the most robust clustering method for analysing noisy expression profile data compared to the other three methods assessed.


**Comparative and Functional Genomics**
*Comp Funct Genom* 2003; **4**: 287–299.
Published online in Wiley InterScience (www.interscience.wiley.com). **DOI:** 10.1002/cfg.290

**Primary Research Paper**

### Performance assessment of kernel density clustering for gene expression profile data

Guoping Shu*, Beiyan Zeng, Yiping P. Chen and Oscar H. Smith
*Reid Research Centre, Pioneer Hi-Bred International Inc., DuPont Agriculture and Nutrition, 7300 NW 62nd Avenue, PO Box 1004, Johnston, IA 50131, USA*

**Correspondence to: Guoping Shu, Reid Research Centre, Pioneer Hi-Bred International Inc., DuPont Agriculture and Nutrition, 7300 NW 62nd Avenue, PO Box 1004, Johnston, IA 50131, USA. E-mail: Guoping.Shu@Pioneer.com*

### Abstract

**Kernel density smoothing techniques have been used in classification or supervised learning of gene expression profile (GEP) data, but their applications to clustering or unsupervised learning of those data have not been explored and assessed. Here we report a kernel density clustering method for analysing GEP data and compare its performance with the three most widely-used clustering methods: hierarchical clustering, K-means clustering, and multivariate mixture model-based clustering. Using several methods to measure agreement, between-cluster isolation, and within-cluster coherence, such as the Adjusted Rand Index, the Pseudo $F$ test, the $r^2$ test, and the profile plot, we have assessed the effectiveness of kernel density clustering for recovering clusters, and its robustness against noise on clustering both simulated and real GEP data. Our results show that the kernel density clustering method has excellent performance in recovering clusters from simulated data and in grouping large real expression profile data sets into compact and well-isolated clusters, and that it is the most robust clustering method for analysing noisy expression profile data compared to the other three methods assessed. Copyright © 2003 John Wiley & Sons, Ltd.**

Received: 20 November 2003
Revised: 24 February 2003
Accepted: 26 February 2003

**Keywords:** **clustering analysis; kernel density; smoothing; gene expression; expression profile; unsupervised learning; robustness; noisy data; pseudo $t$ test; $r^2$; Pseudo $F$; Rand Index**

### Introduction

Various types of genome-wide gene expression profiling experiments have been conducted to measure the differential expression of a large number of genes (Lockhart and Winzeler, 2000; Schena, 2000). Clustering these genes into groups of similar expression profiles is the first step in discovering their biological functions. A number of clustering methods for analysing large gene expression profile (GEP) data have been reported, such as hierarchical clustering (Eisen *et al.*, 1998), K-means clustering (Tavazoie *et al.*, 1999), self-organizing maps (Tamayo *et al.*, 1999), neural networks (Herrero *et al.*, 2001), graph-theoretic clustering (Ben-Dor and Yakhini, 1999; Hartuv *et al.*, 1999), support vector machines (Brown *et al.*, 2000), quality-based clustering (Heyer *et al.*, 1999; De Smet *et al.*, 2002) and multivariate mixture model-based clustering (Yeung *et al.*, 2001; Ghosh and Chinnaiyan, 2002). Here we report a kernel density clustering method for gene expression profile analysis. Kernel density classification and discrimination or supervised learning of GEP data analyses have been reported (Hastie *et al.*, 2001; Li *et al.*, 2001) but kernel density clustering or unsupervised learning of gene expression data have not been explored and assessed.

The kernel density clustering method we report here assumes no parametric statistical models and does not rely on any specific probability distribution. Thus, it is particularly suitable to clustering gene expression patterns from data collected in large gene expression profiling experiments where

Copyright © 2003 John Wiley & Sons, Ltd.

non-Gaussian distribution, heterogeneous variance and complex statistical dependence among variables (e.g. among different tissues and different sampling time points) are the norm.

In this work, we assess the performance and robustness of the kernel density clustering method on grouping simulated and real GEP data, and compare it against the benchmark methods: average-linkage, K-means and multivariate mixture model-based clustering. Our results show that the kernel density clustering method performs among the best and is the most robust method against noise in data.

### Methods

### Kernel density estimation, smoothing and clustering

For a set of observations obtained from a univariate distribution, the oldest and the most widely used probability density estimator is the histogram. Taking any observation $X_i$ as an origin, and a bin or window of width $h$, which is defined as the intervals $[X_i + mh, X_i + (m+1)h]$ for a positive or negative integer $m$, the histogram is defined by:

$$\hat{f}(x) = \frac{1}{nh}(\text{number of observatons } X_i \text{ in the same bin as } x) \quad (1)$$

The above function can be generalized into a naive probability density estimator (Silverman, 1986):

$$\hat{f}(x) = \frac{1}{nh}\sum_{i=1}^{n} w\left(\frac{x - X_i}{h}\right) \quad (2)$$

Where $n$ is the total number of observations in a data set and $w$ is a weight function. The window width $h$ in (2) is also called the smoothing parameter because it controls the amount of smoothing inherent in the density estimation procedure. The density estimate is obtained by placing a 'box' of width $2h$ and height $(2nh)^{-1}$ on each observation and then summing. The naive estimator can be further generalized as a continuous function by replacing the weight function $w$ with a kernel function $K$. The kernel probability density estimator is then defined as:

$$\hat{f}(x) = \frac{1}{nh}\sum_{i=1}^{n} K\left(\frac{x - X_i}{h}\right) \quad (3)$$

Just as the naive estimator can be viewed as a sum of 'boxes' centred at the observations, the kernel estimator is a sum of 'bumps' placed at the observations. The kernel function $K$ determines the shape of the bumps, while the bin, or window, width $h$ determines their widths. Figure 1 illustrates the smoothing effect of two different window widths for a density curve to a histogram. A smaller window width leads to a decrease in smoothing effect and an increase in the number of local maxima (clusters) detected. The definition of (3) can be extended to multivariate data, where the window width $h$ becomes radius of a hypersphere, $R$. The hypersphere specified by $R$ at observation $X_i$ is also called the neighbourhood of $X_i$ in K-nearest neighbourhood density estimation (Silverman, 1986; Scott, 1992). A number of kernel functions have been proposed (Koontz and Fukunaga, 1972; Gitman, 1973; Huizinga, 1978; Wong and Schaack, 1982). We use the hyperspherical uniform kernels of variable radius implemented in SAS (1999). The density estimate at a data point $X_i$ is obtained from dividing the number of observations $n_i$ within a hypersphere centred at the point $X_i$ by the product of the sample size $n$ and the volume of the hypersphere $v_i$, which can be expressed as $\hat{f}_i = n_i/nv_i$. In the SAS implementation, a cluster is defined in terms of the local maxima of a smoothed probability density or a maximal connected set of local maxima of the neighbourhood distribution function (SAS, 1999). The distance or dissimilarity measure between two clusters (or observations) $i$ and $j$ is computed, using:

$$d(x_i, x_j) = \begin{cases} \frac{1}{2}\left(\frac{1}{f(x_i)} + \frac{1}{f(x_j)}\right) & \text{if } d(x_i, x_j) \le R \\ \infty & \text{otherwise} \end{cases} \quad (4)$$

where $R$ is the user-specified radius and $f(x)$ is the estimated density at $x$ (Silverman, 1986; Scott, 1992; SAS, 1999).

To assess both the performance of the kernel density clustering method in discovering cluster structure from profile data and its robustness against noise in data, we compared this method with three other types of clustering methods that are most commonly used in GEP analysis: (a) average-linkage clustering, a hierarchical Euclidean distance-based clustering algorithm (Gordon, 1999; SAS, 1999); (b) adaptive K-means

Copyright © 2003 John Wiley & Sons, Ltd. 

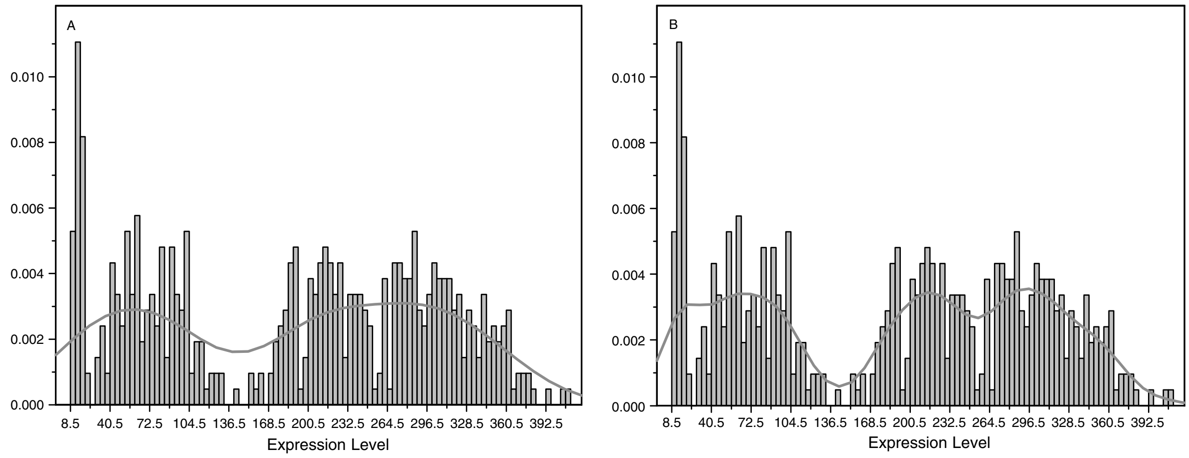

**Figure 1.** Histograms of 520 genes and the kernel density curve fitted using two different smoothing windows ($h$). (A) $h = 150$; two local maxima (clusters) are detected; (B) $h = 45$; four local maxima (clusters) are detected. Bar interval width $= 5$ is used for the histogram of A and B. Only the gene expression level at one sampling time point (variable) from data set D is plotted to illustrate the relationship between smoothing parameter, window width $h$ (equivalently, the radius $R$ for multivariate data), and the number of local maxima (clusters) of the density curve detected by kernel density clustering method clustering, a partitioning clustering algorithm (Gordon, 1999; SAS, 1999); and (c) multivariate mixture model-based clustering (Fraley and Raftery, 1999; Yeung *et al.*, 2001; Ghosh and Chinnaiyan, 2002). For kernel density clustering, average linkage clustering and K-means clustering, we used an SAS macro that we have developed, which incorporates statistical procedures available in the commercial software SAS Version 8.0 (SAS, 1999). For multivariate mixture model-based clustering, we used the Mclust procedure implemented in the R language by Fraley and Raftery (1999), which is available from **http://www.r-project.org/** and **http://www.stat.washington.edu/fraley/Mclust/soft.shtml**. An almost identical implementation is also available in the commercial software, S-Plus, Version 6 (S-Plus, 2001).

### Assessing performance using simulated signal data

A widely used methodology for performance assessment in the clustering literature is called external validation, i.e. evaluating the performance of a clustering algorithm against external criteria. The external validation we used proceeds as follows: (a) a signal (or signature) data set that has $K$ known clusters is generated using Monte Carlo simulation, each observation (vector) in the data set carrying a cluster membership ID (design ID); (b) the data set is clustered by the clustering method for assessment into $K$ clusters and each observation is assigned a new cluster membership ID, called assigned ID. The degree of agreement or similarity between the assigned IDs and the design IDs is estimated using a match coefficient, called the Hubert–Arabie Adjusted Rand Index (ARI; Hubert and Arabie, 1985; Rand, 1971), which has been shown to perform the best among a number of match coefficients assessed by Milligan and Cooper (1986) and Yeung *et al.*, (2001). The statistical principle and computation of the ARI are summarized as follows: let us consider two partitions of the same data set of $n$ objects (observation vectors) $P_1 = [C_{1i}(i = 1, 2, \ldots), c_1]$ and $P_2 = [C_{2j}(j = 1, 2, \ldots), c_2]$, one from a clustering method for assessment and one from either an external criterion (prior knowledge) or a different clustering method. The resemblance between the two partitions can be assessed using information contained in the $c_1 \times c_2$ cross-classification table $(n_{ij})$, where $n_{ij}$ denotes the number of objects in cluster $i$ of partitioning $P_1$ and cluster $j$ of partitioning $P_2$. For $n$ objects, there are totally $\binom{n}{2}$ distinct pairs and they fall into three different categories or types: Type I, pairs that belong to the same cluster in both partitions, $P_1$ and $P_2$, Type II, pairs that belong to different clusters in both $P_1$ and $P_2$, and Type III, pairs that belong to same cluster

Copyright © 2003 John Wiley & Sons, Ltd. 

in one partition and to a different cluster in the other partition. Type I and Type II pairs are those that agree in the two partitions and Type III are those that disagree. The ARI of Hubert and Arabie (1985), which measures the agreement using pairs of the above three types, is given as:

$$R_{HA} = \frac{\sum_{i=1}^{c_1}\sum_{j=1}^{c_2}\binom{n_{ij}}{2} - \sum_{i=1}^{c_1}\binom{n_{i.}}{2}\sum_{j=1}^{c_2}\binom{n_{.j}}{2}/\binom{n}{2}}{\left[\sum_{i=1}^{c_1}\binom{n_{i.}}{2} + \sum_{j=1}^{c_2}\binom{n_{.j}}{2}\right]\Big/2 - \sum_{i=1}^{c_1}\binom{n_{i.}}{2}\sum_{j=1}^{c_2}\binom{n_{.j}}{2}/\binom{n}{2}} \quad (5)$$

where $n_{i.} = \sum_{j=1}^{c_2} n_{ij}$ and $n_{.j} = \sum_{i=1}^{c_1} n_{ij}$, $c_1$ and $c_2$ are the number of clusters in the two partitions.

The $R_{HA} = 1$ when the two partitions are identical. In our case, this indicates a perfect performance of a clustering method in recovering the known cluster structure from the data, $R_{HA} = 0$, when the partitions are selected at random; this would indicate a complete failure of the clustering method in recovering the known clusters from the data.

### Assessing robustness to noise using noisy simulated data

To assess the impact of noise in a data set on the performance of the density clustering method, we compared the performance of the kernel density method on clustering the signal data sets and clustering the 'signal + noise' data set (see Data sets). By applying the density clustering method (or other clustering method) to both data sets, we generate two partitions, as well as two cluster membership ID assignments. We then estimate the ARI, $R_{HA}$, using formula (5) from the two cluster ID assignments. A smaller $R_{HA}$ value indicates a higher impact of noise on the performance of a clustering method. The robustness of a clustering method against noise is measured by examining the change in the $R_{HA}$ value across five levels of noise. A larger change in $R_{HA}$ value at different noise levels is an indication that the clustering method is sensitive to noise, and thus is not robust. At each noise level, four random samples (replications) of noise data are generated, using different random seeds, four ARIs are then computed and their averages and standard deviations are shown in Table 2.

### Applying kernel density clustering to real data

We further assess the performance of the kernel density clustering by applying it to the analysis of two sets of real GEP data. Because in a real data set, the cluster membership for each gene (object) is unknown, external validation, such as the ARI, cannot be employed for performance assessment. We use two statistical criteria: the Pseudo $F$ test, and the accumulated between-cluster $r^2$ test to assess the performance.

The Pseudo $F$ statistic, also called the Calinski–Harabasz test, was first proposed by Calinski and Harabasz (1974) and is defined as:

$$\textit{Pseudo } F = \frac{\left[\sum_{i=1}^{n}(X_i - \overline{X})^2 - \sum_{k}^{G}\sum_{i=1}^{n_k}(X_i - \overline{X}_k)^2\right]\Big/(G-1)}{\left[\sum_{k}^{G}\sum_{i=1}^{n_k}(X_i - \overline{X}_k)^2\right]\Big/(n-G)} \quad (6)$$

where $n$ is the total number of objects in the data, $n_k$ is the number of objects in cluster $k (k = 1, 2, \cdots, G)$, and $X_i$ and $\overline{X}_k$ are the observation vectors for object $i$ and the centroid (the mean vector) for group $k$, respectively, at any level of cluster joining. The Pseudo $F$ statistic is the best global statistical criterion among the 15 criteria of cluster number determination evaluated by Milligan and Cooper (1985).

The accumulated between-cluster $r^2$, also called the coefficient of determination in the statistical literature, measures the proportion of total variation in the data accounted for by between-cluster variation:

$$r^2 = 1 - \frac{\text{Total within-cluster sum of squares}}{\text{Total sum of squares}} = 1 - \frac{\sum_{k}^{G}\sum_{i=1}^{n_k}(X_i - \overline{X}_k)^2}{\sum_{i=1}^{n}(X_i - \overline{X})^2} \quad (7)$$

As pointed out by Gordon (1999), an objective criterion for the best clustering method is that it should produce clusters that show maximum

Copyright © 2003 John Wiley & Sons, Ltd. 

between-cluster isolation and within-cluster coherence and compactness. The statistical criteria most widely used are the Pseudo $F$ test and the $r^2$ test. We also use a graphical display method, called profile plot (Gordon, 1999), to measure within-cluster coherence and compactness (see Figures 5, 6).

The ARI, Pseudo $F$ and $r^2$ statistic were computed using an SAS Macro that we have developed using the SAS Version 8.0 software (SAS, 1999).

### Data sets

### Simulated signal data

We generated five simulated signal, or signature, data sets (labelled as A, B, C, D, E) using the Monte Carlo simulation method of Kuiper and Fisher (1975). We implemented the method in the S language and generated the data in S-plus Version 6.0 (Chambers, 1998; S-plus, 1998–2001).

Each of the four data sets A, B, C and D was generated based on a different mathematical model and represents and simulates a type of profile pattern commonly observed in real expression profile data. Each data set has 520 rows (genes) and 10 columns (time points, or developmental stages, or variables), and is comprised of eight clusters, with cluster size ranging from 20 to 150 genes. Each panel in Figure 2 shows the profiles of eight clusters for each data set. We also introduced heteroscedasticity into the models by holding the coefficient of variation, $CV = \sigma/\mu$, constant across different time points (variables). Heteroscedasticity is also called heterogeneous error variance among samples in statistics (Milliken and Johnson, 1992; Zar, 1999). The type of heteroscedasticity that we modelled, where the sample error variance ($\sigma^2$) increases with sample mean ($\mu$), is a common phenomenon found in GEP data. We pooled the four data sets to form the fifth data set (data set E). The key features of the five simulated signal data sets are:

- *Data set A:* Models development stage-specific, or cell (tissue) type-specific, expression profiles.

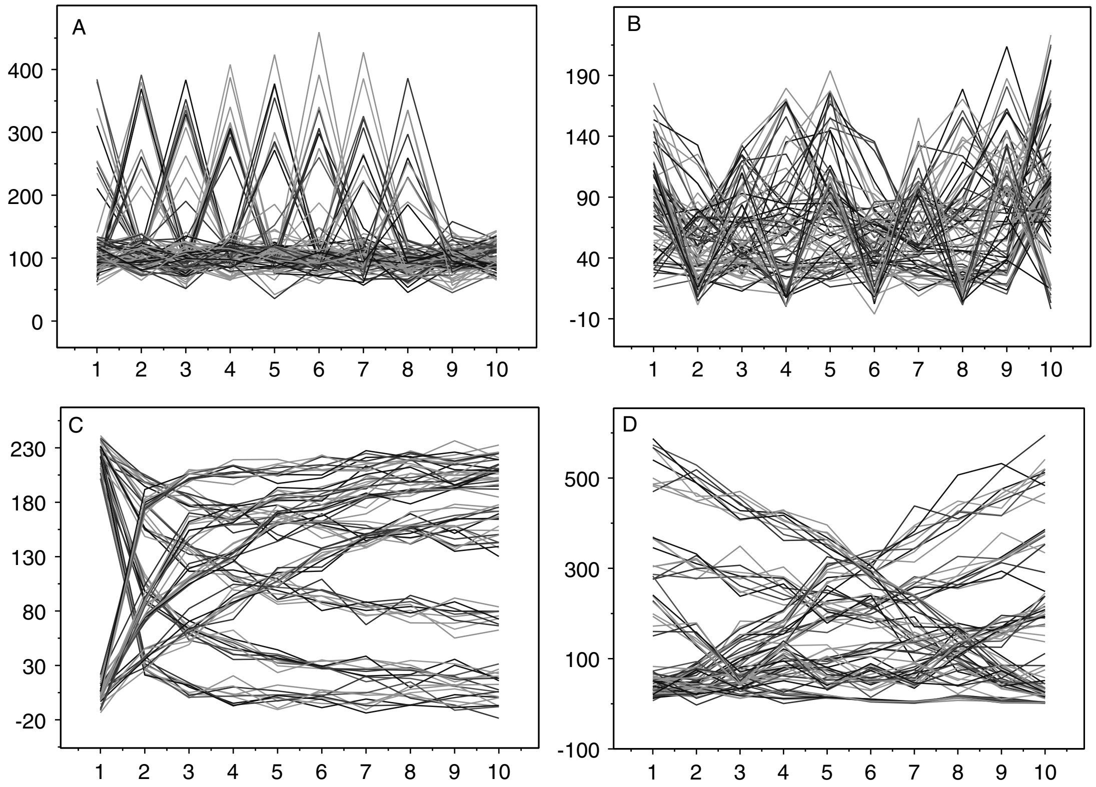

**Figure 2.** Four types of gene expression profiles generated by Monte Carlo simulation. (A) Stage-specific profile; (B) cyclic profile; (C) non-linear profile; (D) linear profile. Each type has eight clusters, only 10 genes per cluster are plotted: *x* axis, 10 sampling time points; *y* axis, intensity (level) of gene expression

Copyright © 2003 John Wiley & Sons, Ltd.

The expression levels of all genes only go up or down once (at one stage) and stay constant across other stages (520 genes, eight clusters).

- *Data set B:* Models cyclic (cell cycle type) expression profiles. The level of gene expression oscillates according to a biological clock, or cell division/organ development cycles (520 genes, eight clusters).
- *Data set C:* Models non-linear patterns of gene expression (520 genes, eight clusters).
- *Data set D:* Models linear or quasi-linear patterns of gene expression (520 genes, eight clusters).
- *Data set E:* Pool of data sets A, B, C and D (2080 genes, 32 clusters).

#### Simulated noise data

Noise data that represent five levels of variation specified by the coefficient of variation ($CV = 0.2$, 0.6,1.0, 1.4 and 1.8, respectively) were generated from the normal distribution $N(\mu, \sigma)$ using the Monte Carlo simulation. At each noise level, a data set of 400 genes, comprising four subsets of 100 genes each, was generated. Each subset takes one of the four values for location parameter ($\mu$), 20, 60, 100 and 160, so that the range of variation in the noise data is comparable to that in the signal data for effective interference. The dispersion parameter $\sigma$ is specified based on the same heteroscedastic variance models used for generating the signal data sets. The key feature that distinguishes the noise data from the signal data is that for each subset of noise data (equivalent to a cluster in a signal data set), the values of $\mu$ and $\sigma$ are held constant across variables (different time points), whereas for each cluster in the signal data set, the values of both parameters change according to a specified mathematical model. The variation patterns of the four noise data sets that represent four noise levels are shown in Figure 3. At each noise level, four data sets that represent four random samples, or replications (rep1, 2, 3, 4), were generated from the same error models using different random seeds. The data sets shown in Figure 2 are all from replication 1.

#### Real gene expression profile data

Two real gene expression profile data sets, generated using microarray technology, were used to evaluate the kernel density clustering method:

- *Data set 1* was collected in Antoni Rofalski's lab at DuPont. The data set we used for this analysis has 1130 genes or ESTs, and five variables, corresponding to five sampling time points during maize (*Zea mays)* embryo development (5, 10, 15, 20, 25 days after pollination; DAP). See Lee *et al.* (2002) for details on the data collection and annotation.
- *Data set 2* was collected from a diauxic shift experiment on the yeast *Saccharomyces cerevisiae* by DeRisi *et al.* (1997). The subset of this data that was used in this analysis contains microarray measures of RNA intensity for 2500 genes at seven sampling time points (variables) after 9 h initial growth in sugar-rich medium (9, 11, 13, 15, 17, 21 h). See DeRisi *et al.* (1997) for further details.

Both data sets were standardized using variable (or column) arithmetic means and standard deviations before undertaking the clustering analysis.

### Results

### Performance in detecting known clusters from simulated data

To assess the performance of the kernel density clustering method on clustering expression profile data, we applied kernel density clustering to four simulated signal data sets (A, B, C, D) that represent four different types of profiles commonly observed in gene expression profiling experiments (Figure 2) and a combination of the four data sets (data set E) (see Data sets for more details). The ARI, which measures the agreement between the cluster membership assigned by the kernel density method and the cluster membership designed or specified by the data generation model, was computed for each simulated data set and reported in Table 1. To assess the relative merit of this method over the clustering methods widely used in profile data analysis, we also computed the ARIs for three other clustering methods: average linkage, K-means, and mixture model-based clustering. For mixture model-based clustering, we assessed the performances of all six mixture models (EI, VI, EEE, VVV, EEV and VEV) under two noise settings [with ($T$) or without ($F$) Poisson noise], implemented in the Mclust software package of Fraley and Raftery (1999) (see Methods and legend

Copyright © 2003 John Wiley & Sons, Ltd. 

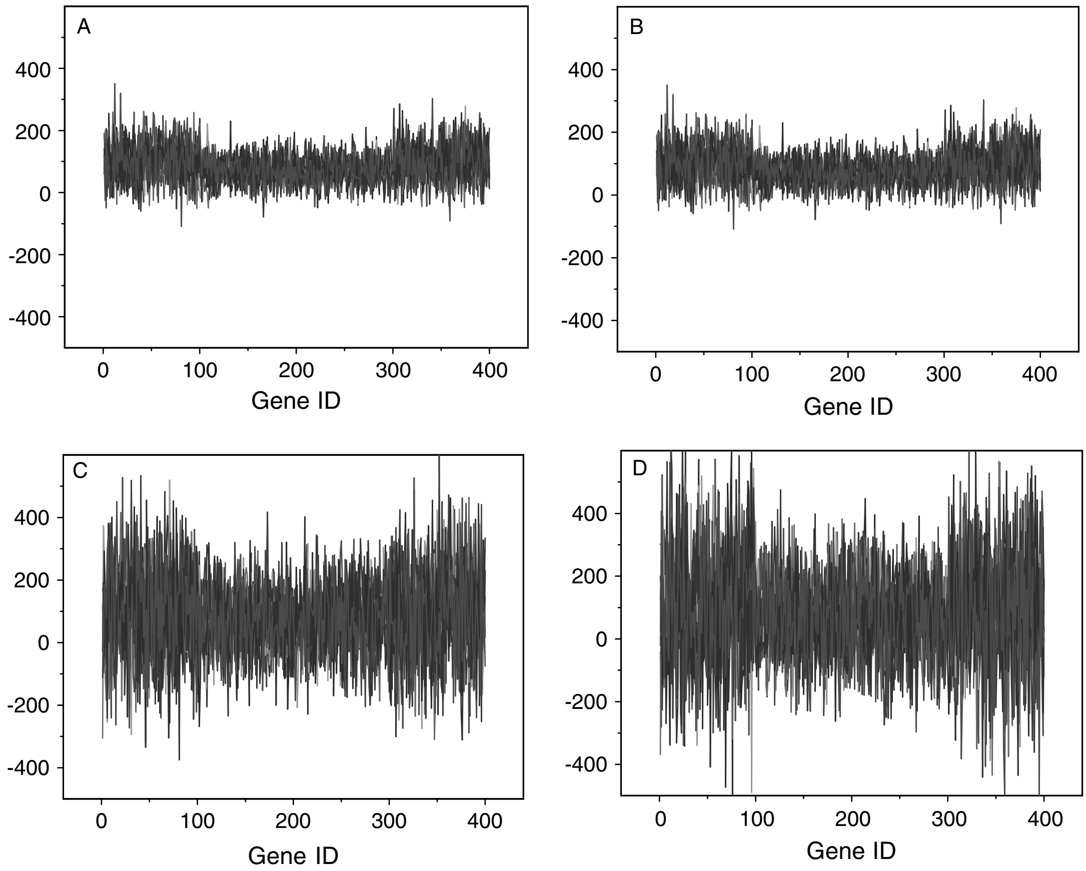

**Figure 3.** Profiles of noise data from replication 1 generated by Monte Carlo simulation. Four different levels of noise measured by the coefficient of variation (CV) are: (A) data set 1, CV = 0.2; (B) data set 2, CV = 0.6; (C) data set 3, CV = 1.0; (D) data set 4, CV = 1.4: *x* axis is the ID for 400 genes from each data set and *y* axis is the range of variation in intensity across 10 time points to Table 1). The VEV and EI models showed the best performance, and are reported in Tables 1 and 2.

For clustering the simulated data of single profile type (data sets A, B, C, D), the results in Table 1 show that the kernel density clustering method performed better than all of the other clustering methods except for VEV-F in identifying clusters from non-linear profiles (C) and linear profiles (D). It is more efficient than the K-means method and the mixture model-based EI-T method, but is marginally less efficient than the average linkage method and the mixture model EI-F, VEV-F, and VEV-T methods in finding clusters from stage-specific profiles (A) and cyclic profiles (B).

For clustering the combined data (data set E), the results in Table 1 show that both the kernel density method and the mixture VEV-F method perform better than average linkage, K-means and all the mixture model-based clustering methods. Since real expression profile data sets are likely to contain profiles of all four types, as demonstrated in Figure 5, the ARIs from data set E are a more reliable indicator of the overall performance of a clustering method. Therefore, the results in Table 1 indicate that the kernel density method has excellent overall performance for clustering of the simulated expression profile data. Table 1 also shows that the average linkage method performs very poorly on clustering the combined data set E (ARI = 0.07), although it performs well in clustering the four data sets consisting of single-profile types. The mixture model-based clustering methods without assuming Poisson noise (VEV-F, EI-F) perform better than those assuming Poisson noise (VEV-T, EI-T; Table 1).

Copyright © 2003 John Wiley & Sons, Ltd. 

**Table 1.** Performance of four clustering methods on clustering four types of simulated expression profile data (ARI* between designed and assigned cluster IDs)

| Data set | Clusters | Kernel density | K-means | Average linkage | Mixture (EI-F) | Mixture (EI-T) | Mixture (VEV-F) | Mixture (VEV-T) |
|---|---|---|---|---|---|---|---|---|
| A (Stage-specific) | 8 | 0.86 | 0.78 | 1.00 | 1.00 | 0.32 | 0.99 | 0.94 |
| B (Cyclic) | 8 | 0.75 | 0.70 | 0.92 | 0.85 | 0.28 | 0.77 | 0.83 |
| C (Non-linear) | 8 | 0.89 | 0.88 | 0.60 | 0.58 | 0.77 | 0.98 | 0.91 |
| D (Linear) | 8 | 0.91 | 0.78 | 0.79 | 0.74 | 0.24 | 1.00 | 0.81 |
| E (Combined) | 32 | 0.85 | 0.76 | 0.07 | 0.81 | 0.11 | 0.89 | 0.56 |

* ARI, adjusted Rand index; EI and VEV are two mixture models; *F*, noise = false; T, noise = true; see Methods section for detail.

**Table 2.** Robustness of four clustering methods against noise in expression profile data (ARI* between assigned cluster IDs from signal data and signal + noise data)

| Noise level | CV | Kernel density | K-means | Average linkage | Mixture (EI-F) | Mixture (EI-T) | Mixture (VEV-F) | Mixture (VEV-T) |
|---|---|---|---|---|---|---|---|---|
| 1 | 0.2 | 0.95 (0.026) | 0.71 (0.027) | 0.99 (0.0008) | 0.97 (0.042) | 0.83 (0.065) | 0.87 (0.055) | 0.66 (0.037) |
| 2 | 0.6 | 0.98 (0.013) | 0.69 (0.020) | 0.98 (0.0063) | 0.94 (0.031) | 0.64 (0.10) | 0.90 (0.037) | 0.54 (0.072) |
| 3 | 1.0 | 0.99 (0.0069) | 0.62 (0.060) | 0.63 (0.052) | 0.84 (0.067) | 0.60 (0.071) | 0.87 (0.094) | 0.36 (0.13) |
| 4 | 1.4 | 0.99 (0.0068) | 0.59 (0.037) | 0.53 (0.099) | 0.93 (0.048) | 0.52 (0.054) | 0.91 (0.032) | 0.47 (0.083) |
| 5 | 1.8 | 0.99 (0.0051) | 0.56 (0.047) | 0.52 (0.096) | 0.82 (0.094) | 0.51 (0.051) | 0.90 (0.081) | 0.46 (0.098) |

* ARI, adjusted Rand index; CV, coefficient of variation; see Table 1 legend for EI, VEV, F, T.
The data in this table are the mean and standard deviation (in parenthesis) computed from four replicated data sets; see Data sets section for more detail.

### Robustness against noise

As many studies have shown, the data from gene expression profiling experiments are usually noisy (Lee *et al.*, 2000; Tseng *et al.*, 2001). In order to systematically assess the robustness of the kernel density clustering method against noise in expression profile data, we examined its performance on clustering simulated signal + noise data. Table 2 shows the average and the standard deviation of the ARI from four replicated data sets generated at every noise level. The results show that the kernel density clustering method is the most robust method and it assigns 95–99% of the 2080 genes into correct clusters at all noise levels. The K-means method performs poorly at all five noise levels. The clustering effectiveness of the average linkage method decreases from 0.99 to 0.52 when the level of noise increases from level 1 to level 5, in contrast to the kernel density method, which performs better at higher noise levels.

We assessed the robustness of all of the six mixture models implemented in the Mclust software package of Fraley and Raftery (1999) (see Methods). Here we only report the results from the two most robust models, the VEV and EI models (Table 2), since the other four models all showed low robustness (0.12–0.68). Table 2 also shows that the mixture models assuming no Poisson noise (EI-F, VEV-F) outperform those assuming Poisson noise (EI-T, and VEV-T) at all five noise levels.

One might have noted that the ARI for the average linkage method on clustering signal data set E is very low (ARI = 0.07; Table 1) but it is very high on clustering signal + noise data (ARI = 1.0 and 0.99 at noise levels 1 and 2, respectively; Table 2). This apparent discrepancy is due to the fact that the ARI in Table 1 is estimated by matching the assigned cluster ID and the design ID of the same signal data. Whereas the ARI in Table 2 is estimated by matching the assigned ID of two data sets: the signal data and the signal + noise

Copyright © 2003 John Wiley & Sons, Ltd. 

data. As pointed out in Methods, the former is suitable for assessing the performance, or the rate of cluster recovery, of a clustering method, and the latter is suitable for assessing the robustness of a clustering method against noise. This computation strategy enables us to assess the performance and robustness independently. The apparent discrepancy in the ARI for the average linkage clustering from Tables 1 and 2 can be explained as follows: the average linkage method is poor in recovering known clusters from the simulated data, but its performance (although it is poor) is less affected by low-level noises (but is strongly affected by high-level noise). Summarizing the results from Tables 1 and 2, we can state that the kernel density clustering method shows excellent performance and robustness, and that the average linkage method is poor, and the K-means method is mediocre in both performance and robustness. The performance and robustness of the mixture model-based clustering methods depend on the models specified; the mixture VEV-F model shows the best overall performance and robustness among all of the mixture models.

### Kernel density clustering of real data

We applied the density clustering method to two real GEP data sets to assess its performance. Since the true number of clusters is unknown for a real data set, the performance of a clustering method in recovering true cluster structure in a real data set cannot be assessed using an external validation method such as the ARI, as reported in the previous section for the simulated data. In addition, because the correlation between the expression profile of a gene (measured by the level of mRNA accumulation in an expression profiling experiment) and the biological function of the gene (measured by its protein activity or/and its physiological and developmental roles) is low and indirect, the performance of a clustering method cannot be assessed accurately by measuring the functional similarity amongst genes within the same cluster. An objective criterion of assessing the performance of a clustering method in real data is to directly measure the observed data (the expression profile in this case) for within-cluster similarity, or coherence, and between-cluster isolation (Gordon, 1999). Here we employ two such statistical criteria, the Pseudo $F$ test and the $r^2$ test, and one graphical inspection method, the profile plot, for this task (see Methods). We can see from Figure 4A that for the kernel density method, there are three local maxima of the Pseudo $F$ value ($y$ axis) at 9, 14, and 17 clusters ($x$ axis). The $r^2$ value reaches 0.76 at 17 clusters, an indication that 76% of total variation in the data can be explained as between-cluster variation, when clustered into 17 clusters by the kernel density method. We inspected the compactness of each cluster and the within-cluster coherence using a profile plot, and the results for 17 clusters are shown in Figure 5. We also applied the kernel density clustering method to the analysis of the yeast diauxic shift data (see Data sets). The Pseudo F and $r^2$ value in Figure 4B indicate that partitioning the data into seven or eight clusters is parsimonious. Figure 6 shows the profile of

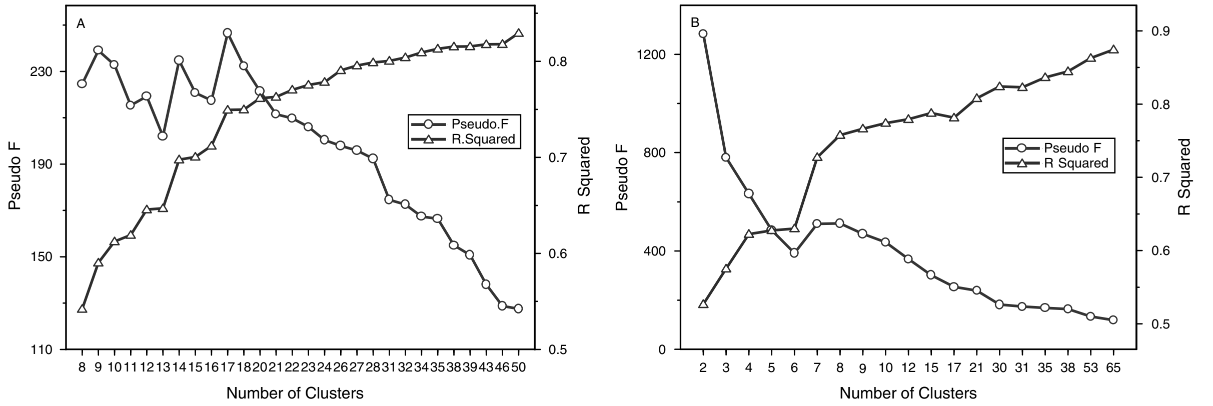

**Figure 4.** Pseudo F and $r^2$ values at different cluster cutoffs (partitions) for two sets of real gene expression profile data. (A) Maize embryo development microarray data; (B) yeast diauxic shift microarray data

Copyright © 2003 John Wiley & Sons, Ltd. 

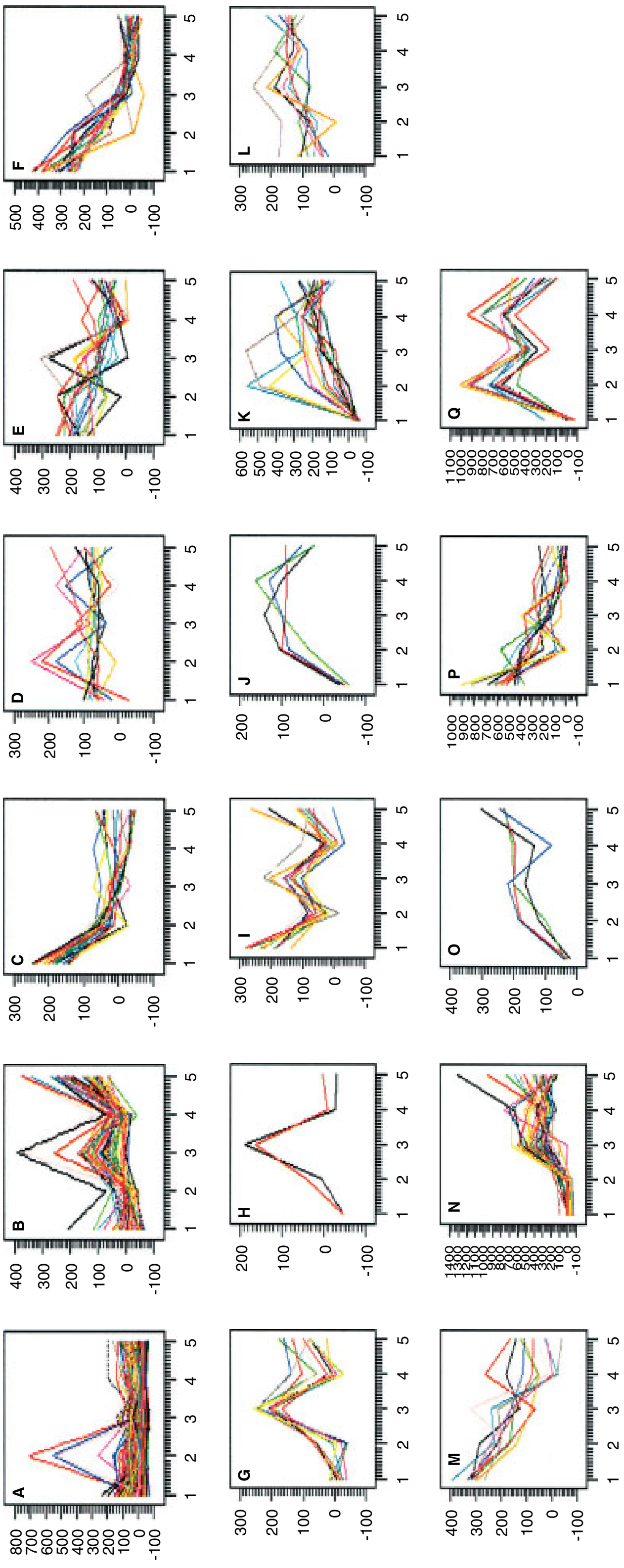

**Figure 5.** Profile plots of 17 clusters detected from the maize embryo-development microarray data: *x* axis, sampling time point; *y* axis, level of expression

Copyright © 2003 John Wiley & Sons, Ltd.

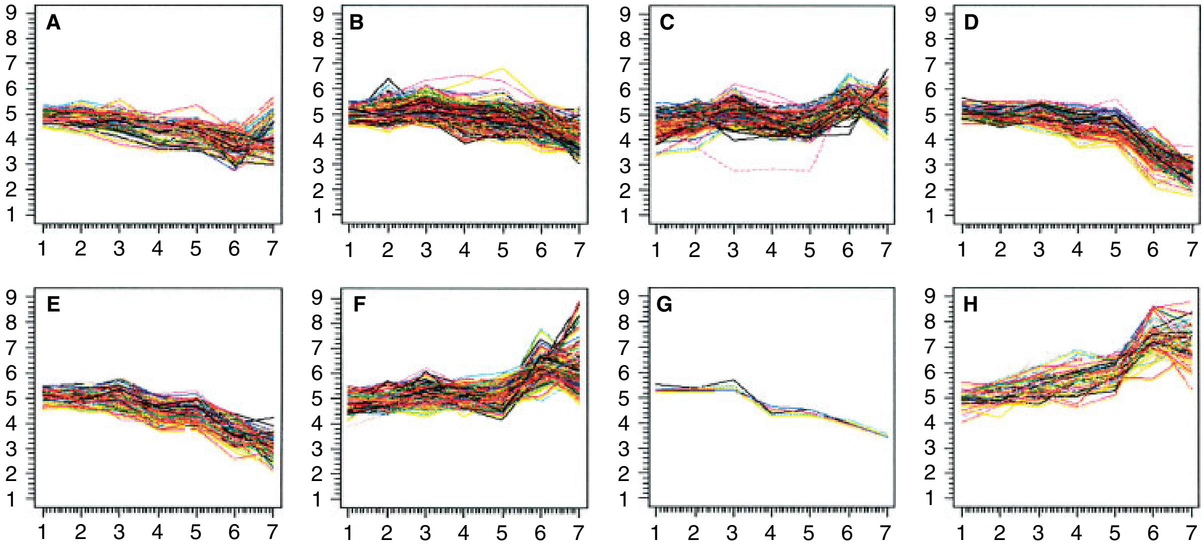

**Figure 6.** Profile plot of eight clusters in the yeast diauxic shift microarray data: *x* axis, sampling time point; *y* axis, level of expression each cluster when the data is partitioned into eight clusters.

We compared our clustering result with the experimental results of DeRisi *et al.* (1997). The authors classify the 35 genes that they studied into five groups, based on their shared regulatory properties in metabolic pathways and their temporal expression profiles. Seventeen of these genes are also present in the 2500 genes that we used for clustering (data set 2 in the Data sets section). Our kernel density clustering assigns the 17 genes into five clusters, which completely agree with the five groups of DeRisi *et al.* (ARI = 1).

The profile plots in Figures 5 and 6 show that the kernel density clustering method performs very well, that the clusters produced by this method are compact and coherent, and that genes with similar trends, or a similar level of abundance, across time points are grouped into the same cluster. We can see from Figure 5 that all of the four different types of profiles that we have modelled in the simulated data (Figure 2) are present in the real expression profile data. They are the stage-specific or time point-specific profile (A, H), the cyclic profile (B, I and Q), the non-linear profile (J, L and N), and the linear or quasi-linear profile (C, F and P). The genes that have quite similar trends across time points, but different levels of abundance of mRNA accumulation, such as C, F and H in Figure 5, and D and E in Figure 6, are grouped into different clusters.

### Discussion

Here we report a kernel density clustering method for analysis of GEP data and assess its performance and robustness on simulated and real expression profile data. The results from the simulated data demonstrate that the kernel density method has excellent performance and is the most robust method against noise in the data. The results from real expression profile data show that the kernel density method can group genes into compact, coherent clusters from large data sets. Therefore, this method should be considered seriously by researchers and data analysts when grouping genomic data. Our results show that the kernel density method is the most robust method for clustering noisy data among the four types of methods we have assessed. Robustness is important in gene-expression profile data analysis because data from many experiments usually reside in a large interactive database and large variations in the scale and quality of data are common. A clustering method that is less sensitive to noise and that requires less data preprocessing to remove or accommodate scale differences will be more useful. Robustness is also important, due to the fact that the majority of genes spotted on a gene chip would not

Copyright © 2003 John Wiley & Sons, Ltd. 

show real change or real differential expression in any specific treatment-control experiment and the observed changes in chip readouts from these genes are largely noise, resulting from sampling, experimental, and measurement errors. This noise would severely affect the performance of a clustering method that is less robust.

One important difference between the kernel density clustering method and the other three clustering methods is that users do not specify the number of clusters before a clustering run (K-means clustering, mixture model-based clustering) or after a clustering run (average linkage clustering). The users instead specify a smoothing parameter, and the program finds the optimum number of clusters. Because the clustering outcome is influenced in some degree by the value of the smoothing parameter specified by the user, as illustrated in Figure 1, several different values for $R$ should be examined in test runs to identify the optimum value of the smoothing parameter for the final clustering run. In most cases, we find that an $R$-value between 0.15 and 1.2 gives a satisfactory clustering result. Mixture model-based clustering implemented in the Mclust package of Fraley and Raftery (1999) also allows users to obtain the optimum cluster number $K$ using a two-step procedure (Fraley and Raftery, 1999); the users run a clustering analysis at every cluster number cutoff ($K$) first, and then identify the optimum $K$ for the final run, based on the value of the Bayesian Information Criterion (BIC). Our results show that the mixture model-based VEV-F method of Fraley and Raftery (1999) has excellent performance and robustness for clustering GEP data sets, although the computational speed becomes much slower than for the kernel density method, K-means, and average linkage clustering when the size of a data set is large.

We found that the kernel density clustering method is particularly suitable for clustering large data sets. For instance, when clustering thousands of genes in a large microarray data set into groups, the average linkage method is overwhelmed by outlier genes and tends to lose power in finding true clusters at a lower cluster number cut-off ($K$); the K-means and model-based EI methods tend to find clusters with roughly the same number of observations (genes); the mixture model-based clustering methods become very slow; but the kernel density clustering method is faster and has more partitioning power for finding clusters of various sizes than all of the above three clustering methods. In our opinion, the kernel density method's flexibility, speed and robustness are properties that make it a promising method for clustering large GEP data sets.

Like other clustering methods, the kernel density clustering method has limitations. Because accurate estimation of density and assignment of cluster membership require multiple data points in near neighbourhoods, density estimation is less accurate when a cluster size (expected number of observations in a cluster) is very small. However, this is also why this method is robust and less sensitive to outliers.

Since the main focus of this work is to introduce a new clustering method to the bioinformatics and genomics community and to demonstrate its performance and utility for clustering expression profile data, rather than systematically comparing the relative merits of the different types of clustering methods, the number of clustering methods that were used as benchmarks was limited, and we did not assess the self-organizing maps method (Tamayo *et al.*, 1999) and the quality-based clustering methods (Heyer *et al.*, 1999; De Smet *et al.*, 2002), for example. For the same reasons, the scope of our simulation study for assessing the performance and robustness of different clustering procedures is also relatively small. Since it has been well documented in the clustering literature that different clustering methods are suitable for different types of data (Gordon, 1999), the performance and robustness of the kernel density clustering method on clustering other types of genomic data will need further assessment.

### Acknowledgements

We would like to thank Antoni Ralfaski for providing the maize embryo microarray data and Patrick Brown for the Yeast diauxic shift microarray data. We would also like to thank Chris Fraley and Murua Raftery for the mixture model-based clustering algorithm, Mclust. Our thanks also go to our colleagues Mark Cooper and Kevin Wright for many helpful discussions.

### References

Banfield JD, Raftery AE. 1993. Model-based Gaussian and non-Gaussian clustering. *Biometrics* **49**: 803–821.

Copyright © 2003 John Wiley & Sons, Ltd. 

Ben-Dor A, Yakhini Z. 1999. Clustering gene expression patterns. In *RECOMB99: Proceedings of the Third Annual International Conference on Computational Molecular Biology*, Lyon, France.

Brown MPS, Grundy WN, Lin D, *et al*. 2000. Knowledge-based analysis of microarray gene expression data using support vector machines. *Proc Natl Acad Sci USA* **97**: 262–267.

Calinski T, Harabasz J. 1974. A dendrite method for cluster analysis. *Commun Statist* **3**: 1–27.

Chambers JM. 1998. *Programming with Data. A Guide to the S Language*. Springer-Verlag: New York.

De Smet F, Mathys J, Marchal K, *et al*. 2002. Adaptive quality-based clustering of gene expression profiles. *Bioinformatics* **18**: 735–746.

DeRisi JL, Vishwanath RL, Brown PO. 1997. Exploring the metabolic and genetic control of gene expression on a genomic scale. *Science* **278**: 680–686.

Eisen MB, Spellman PT, Brown PO, Botstein D. 1998. Cluster analysis and display of genome-wide expression patterns. *Proc Natl Acad Sci USA* **95**: 14 863–14 868.

Fraley C, Raftery AE. 1999. Mclust: software for model-based cluster analysis. *J Classif* **16**: 297–306.

Ghosh D, Chinnaiyan AM. 2002. Mixture modelling of gene expression data from microarray experiments. *Bioinformatics* **18**: 275–286.

Gitman I. 1973. An algorithm for nonsupervised pattern classification. *IEEE Trans Syst Man Cybernet* **SMC-3**: 66–74.

Gordon AD. 1999. *Classification*, 2nd edn. Chapman & Hall/CRC: London.

Hartuv E, Schmitt A, Lange J, *et al*. 1999. An algorithm for clustering cDNAs for gene expression analysis. In *RECOMB99: Proceedings of the Third Annual International Conference on Computational Molecular Biology*, Lyon, France.

Hastie T, Tibshirani R, Friedman J. 2001. *The Elements of Statistical Learning: Data Mining, Inference, and Prediction*. Springer-Verlag: New York.

Herrero J, Valencia A, Dopazo J. 2001. A hierarchical unsupervised growing neural network for clustering gene expression patterns. *Bioinformatics* **17**: 126–136.

Heyer LJ, Kruglyak S, Yooseph S. 1999. Exploring expression data: identification and analysis of co-expressed genes. *Genome Res* **9**: 1102–1115.

Hubert L, Arabie P. 1985. Comparing partitions. *J Classif* **2**: 193–218.

Kuiper FK, Fisher L. 1975. A Monte Carlo comparison of six clustering procedures. *Biometrics* **31**: 777–783.

Huizinga DH. 1978. *A Natural or Mode Seeking Cluster Analysis Algorithm*. Technical Report 78-1. Behavioral Research Institute: 2305 Canyon Blvd., Boulder, CO 80 302.

Koontz WLG, Fukunaga K. 1972. A non-parametric valley-seeking technique for cluster analysis. *IEEE Trans Comput* **C-21**: 171–178.

Lee J, Williams ME, Tingey SV, Rafalski JA. 2002. DNA array profiling of gene expression changes during maize embryo development. *Funct Integr Genom* **2**: 13–27.

Lee M-LT, Kuo FC, Whitmore GA, Sklar J. 2000. Importance of replication in microarray gene expression studies: statistical methods and evidence from repetitive cDNA hybridization. *Proc Natl Acad Sci USA* **97**: 9834–9839.

Li L, Weinberg CR, Darden TA, Pedersen LG. 2001. Gene selection for sample classification based on gene expression data: study of sensitivity to choice of parameters of the GA/KNN method. *Bioinformatics* **17**: 1131–1142.

Lockhart DJ, Winzeler EA. 2000. Genomics, gene expression and DNA arrays. *Nature* **405**: 827–836.

Milligan GW, Cooper MC. 1985. An examination of procedures for determining the number of clusters in a data set. *Psychometrika* **50**: 159–179.

Milligan GW, Cooper MC. 1986. A study of the comparability of external criteria for hierarchical cluster analysis. *Multivar Behav Res* **21**: 441–458.

Milliken GA, Johnson DE. 1992. *Analysis of Messy Data, vol I: Designed Experiments*. Chapman & Hall: London.

Rand WM. 1971. Objective criteria for the evaluation of clustering methods. *J Am Statist Assoc* **66**: 846–850.

*S-PLUS 6.0 for Windows*, Professional Release 1, Copyright © 1988–2001. Insightful Corp.

*SAS/STAT User's Guide*, Version 8.0, 1999. SAS Institute Inc: Cary, NC.

Schena M. 2000. *Microarray Biochip Technology*. Eaton: Natick, MA.

Schwarz G. 1978. Estimating the dimension of a model. *Ann Stat* **6**: 461–464.

Scott DW. 1992. *Multivariate Density Estimation: Theory, Practice, and Visualization*. Wiley: New York.

Silverman BW. 1986. *Density Estimation*. Chapman & Hall: New York.

Tamayo P, Slonim D, Mesirov J, *et al*. 1999. Interpreting patterns of gene expression with self-organizing maps: methods and application to hematopoietic differentiation. *Proc Natl Acad Sci USA* **96**: 2907–2912.

Tavazoie S, Hughes JD, Campbell MJ, Cho RJ, Church GM. 1999. Systematic determination of genetic network architecture. *Nature Genet* **22**: 281–285.

Tseng GC, Oh M-K, Rohlin L, Liao JC, Wong WH. 2001. Issues in cDNA microarray analysis: quality filtering, channel normalization, models of variations and assessment of gene effects. *Nucleic Acids Res* **29**: 2549–2557.

Wong MA, Schaack C. 1982. Using the $K$th nearest neighbor clustering procedure to determine the number of subpopulations. *Proceedings of the Statistical Computing Section*. American Statistical Association: 1982; 40–48.

Yeung KY, Fraley C, Murua A, Raftery AE, Ruzzo WL. 2001. Model-based clustering and data transformations for gene expression data. *Bioinformatics* **17**: 977–987.

Zar JH. 1999. *Biostatistical Analysis*, 4th edn. Prentice Hall: Upper Saddle River, NJ.

Copyright © 2003 John Wiley & Sons, Ltd. 
